# Dataset of milk whey proteins of three indigenous Greek sheep breeds

**DOI:** 10.1016/j.dib.2016.06.040

**Published:** 2016-06-29

**Authors:** Athanasios K. Anagnostopoulos, Angeliki I. Katsafadou, Vasileios Pierros, Evangelos Kontopodis, George C. Fthenakis, George Arsenos, Spyridon Ch. Karkabounas, Athina Tzora, Ioannis Skoufos, George Th. Tsangaris

**Affiliations:** a Proteomics Research Unit, Center of Basic Research II, Biomedical Research Foundation of the Academy of Athens, Athens, Greece; b Veterinary Faculty, University of Thessaly, Karditsa, Greece; c Laboratory of Animal Husbandry, School of Veterinary Medicine, Aristotle University of Thessaloniki, Thessaloniki, Greece; d Cell and Molecular Physiology Unit, Laboratory of Experimental Physiology, Medical School, University of Ioannina, Ioannina, Greece; e Department of Animal Production, Technological Educational Institute of Epirus, Arta, Greece

## Abstract

The importance and unique biological traits, as well as the growing financial value, of milk from small Greek ruminants is continuously attracting interest from both the scientific community and industry. In this regard the construction of a reference dataset of the milk of the Greek sheep breeds is of great interest. In order to obtain such a dataset we employed cutting-edge proteomics methodologies to investigate and characterize, the proteome of milk from the three indigenous Greek sheep breeds Mpoutsko, Karagouniko and Chios. In total, more than 1300 protein groups were identified in milk whey from these breeds, reporting for the first time the most detailed proteome dataset of this precious biological material. The present results are further discussed in the research paper “Milk of Greek sheep and goat breeds; characterization by means of proteomics” (Anagnostopoulos et al. 2016) [1].

**Specifications Table**

**Table:** 

Subject area	*Foodomics, Veterinary science*
More specific subject area	*Mpoutsko, Karagouniko and Chios sheep milk proteome*
Type of data	*Excel file, Figure*
How data was acquired	1D-nanoLC-MS/MS, bottom-up proteomics
	Dionex Ultimate 3000 nanoHPLC system coupled to an LTQ Velos *Orbitrap Elite mass spectrometer* (Thermo Scientific, Rockford, IL, USA)
	PepMap® RSLC, C18, 100 Å, 3 μm-bead-packed 15 cm column and 2 μm-bead-packed 50 cm column (Thermo Scientific)
	Proteome Discoverer 1.4 software (Thermo Scientific), Sequest search engine searching the *Ovis aries* *.fasta databases for milk of sheep.
Data format	*Analyzed*
Experimental factors	*Milk samples from the indigenous Greek sheep breeds Mpoutsiko, Karagouniko and Chios, were systematically collected and analyzed in order to characterize the protein content of the milk of each breed.*
Experimental features	*Whole-proteome analysis of milk whey*
Data source location	*Athens, Greece*
Data accessibility	*Datasets are directly provided with this article*

**Value of the data**

• To report for the first time the proteome dataset of the milk whey from the three indigenous Greek sheep breeds.
• Data could used to govern future steps in optimizing characteristics and features of sheep milk products.
• Data could be used for the traceability of the dairy products of these breeds.
• The comparative analysis of the data could lead to new dairy products with specific nutritional characteristics for the human health.

## 1 Data

**Fig. 1:**
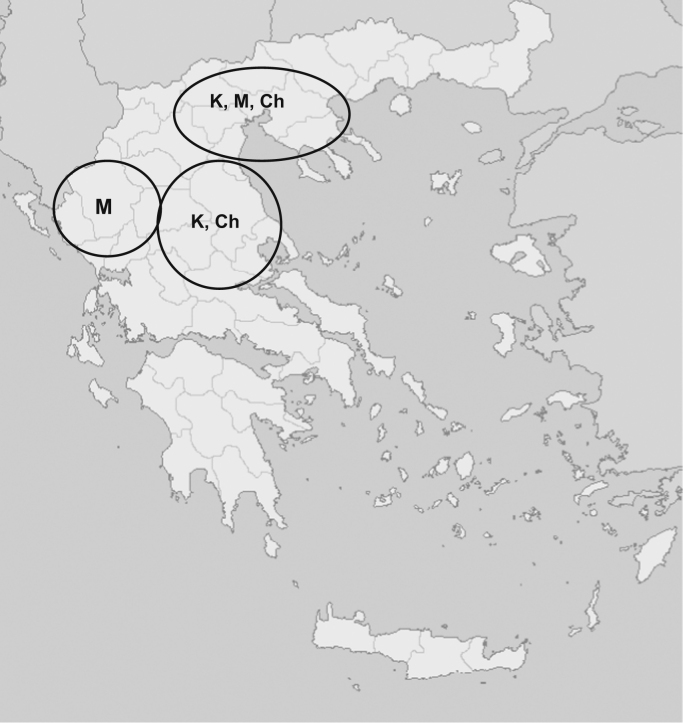
Geographical distribution of Greek sheep flocks used for milk sample collection and proteomic analysis. The three breeds of indigenous Greek sheep (K, Karagkouniko; M, Mpoutsko and Ch, Chios) included in the study can be seen scattered throughout Greece.

Milk samples of the Mpoutsko, Karagouniko and Chios indigenous Greek sheep breeds, were collected by milking at three different time points, throughout the lactation period, on various farms and flocks in regions scattered across Greece (Fig. 1). In total 1303 proteins were identified in the studied samples (Table 1). More specifically, the milk proteome lists comprised of 550 protein groups for the Karagouniko breed, 583 for the Mpoutsko breed and 685 protein groups for the Chios breed.

## 2 Experimental design, materials and methods

### 2.1 Animals and sample collection

Approximately 20 mL of milk collected from the pure-breed sheep Mpoutsko, Karagouniko, and Chios breed, aged 3 to 5 years and 49 to 63 kg in weight, were used in the study. Samples were analyzed as previously reported [1]. All animal procedures regarding animal care and use were approved by the Ethical Committee of the Faculty of Veterinary Medicine, School of Health Sciences, University of Thessaly (Karditsa, Greece).

### 2.2 Sample preparation

Following complete thawing in room temperature, samples were centrifuged at 4000×*g* for 1 h at 4 °C, for final fragmentation in three layers (lipid-, whey-, casein-layer). The flowchart of the strategy followed including the end-process for protein identification approaches used is represented previously [1]. The whey fraction was extracted and the protein content was determined the Bradford assay [2].

### 2.3 Peptide generation and 1-D nano-LC-MS/MS analysis

Protein extraction and peptide generation, was performed as described by our group elsewhere, with few modifications [1], [3]. In brief, 200 ng whey fractions were treated with urea and triethyl ammonium bicarbonate (TEAB) under mild sonication in a water-bath for 30 min and reduction and alkylation steps of proteins were carried out using dithiothreitol and iodoacetamide solutions, The final step of processing included overnight digestion of extracted proteins of all samples by trypsin (Roche Diagnostics, Basel, Swiss) at a final concentration of 500 ng/μl.

Digested samples were analyzed using a LTQ Orbitrap Elite coupled to a Dionex 3000 nanoHPLC system (Thermo Scientific, Rockford, IL, USA). LC separation of peptides took place on two Thermo Scientific columns (PepMap^®^ RSLC, C18, 100 Å, 3 μm-bead-packed 15 cm column and 2 μm-bead-packed 50 cm column) at a flow rate of 3 nL/min. The mobile phases A and B were 0.1% formic acid in water and 99% ACN in water, respectively. The gradient elution profile was as follows: 2.0% B (98.0% A) for 10 min, 2.0–35.0% B (98.0–65.0% A) for 325 min, 80.0% B (20.0% A) for 10 min, 2.0% B (98.0% A) for 10 min. Data were collected in the data-dependent MS/MS mode using a standard top-20 method. Full-scan data were acquired at a resolving power of 60,000 with a maximum integration time of 250 ms. Scan range was fixed at 250–1250 m/z and peptide fragmentation was performed in a higher-energy collision dissociation (HCD) mode with a normalized collision energy of 36%. MS/MS spectra were acquired with 15,000 resolving power and a maximum integration time of 120 ms. Measurements were performed using *m*/*z* 445.120025 as lock mass. Dynamic exclusion settings were set to repeat count 1, repeat duration 30 s, exclusion duration 120 s, and exclusion mass width 0.6 *m*/*z* (low) and 1.6 *m*/*z* (high).

The *.raw data files were analyzed using the Proteome Discoverer software (Thermo Scientific), using the Sequest search engine applying the *Ovis aries* for milk of sheep *.fasta databases. MS/MS searches were performed using a 10 ppm parent ion mass tolerance and a 0.05 fragment mass tolerance. Trypsin was selected as the cleavage enzyme with up to 2 missed cleavage points. Cysteine methylthio modification was selected as a fixed modification and oxidation of methionine were selected as a variable. Peptide identifications were considered valid at 1% False Discovery Rate (*q*-value<0.01) (percolator maximum Delta Cn was 0.05). The minimum length of acceptable identified peptides was set as 6 amino acids.
